# Breast Cancer During Pregnancy and Lactation: A Five-Year Retrospective Study in Sudan

**DOI:** 10.7759/cureus.102646

**Published:** 2026-01-30

**Authors:** Ahmed Aydrose, Hussein E Elsdaig, Adnan Abdalla, Mona Abdalla, Fatima Alsadig, Tartel Ahmed, Esraa Abdallah, Ahmed Alagha, Tarik Yousif

**Affiliations:** 1 Surgery, The National Ribat University, Khartoum, SDN; 2 Surgery, Sudan Medical Specialization Board, Khartoum, SDN; 3 General Internal Medicine, The National Ribat University, Khartoum, SDN; 4 Internal Medicine, University Hospital Limerick, Limerick, IRL; 5 General Surgery, Sudan Medical Specialization Board, Khartoum, SDN; 6 Surgery, Sudan Medical Council, Khartoum, SDN; 7 General Practice, Sudan Medical Council, Khartoum, SDN

**Keywords:** breast cancer research, cancer patients, cross sectional studies, lactation, pregnancy counseling

## Abstract

Introduction

Breast cancer diagnosed during pregnancy or within the first year postpartum is termed pregnancy-associated breast cancer (PABC), and it is the most common malignancy occurring during pregnancy.

Objective

The objective of this study was to investigate the characteristics of breast cancer patients diagnosed during pregnancy and lactation at Wad-Madani Teaching Hospital, Sudan, from 2017 to 2022.

Methods

A retrospective, cross-sectional study was conducted, screening 2,151 breast cancer patients. After applying age and menopausal status exclusions, 33 patients (2.69% of eligible patients) who experienced symptom onset during pregnancy or lactation were included. Data on demographics, clinical presentation, risk factors, receptor status, and histopathology were collected from hospital records.

Results

The most common clinical presentation was a palpable breast lump (31/33, 93.9%), followed by breast pain (12/33, 36.4%) and skin changes or ulceration (10/33, 30.3%). Inflammatory features were present in 11 patients (33.3%). Obesity (9/33, 27.3%) and early menarche (10/33, 30.3%) were prevalent risk factors. Histopathological analysis revealed invasive ductal carcinoma (IDC) in 28 patients (84.8%). Regarding receptor status, 21 patients (63.6%) were estrogen/progesterone receptor (ER/PR)-negative, and 24 patients (72.7%) were human epidermal growth factor-2 (HER2)-negative.

Conclusion

Physiological changes during pregnancy and lactation complicate breast cancer detection and diagnosis. These findings underscore the need for heightened clinical suspicion, early investigation of breast symptoms, and a multidisciplinary approach to improve outcomes in this patient population.

## Introduction

In 2022, 2.3 million women were diagnosed with breast cancer (BC) globally, resulting in 670,000 deaths and establishing it as the most common cancer among women [1]. In many developing countries, BC incidence is rising rapidly, with over half of new cases now occurring in the developing world, where mortality rates are high and overall survival is poor [2]. In Sudan, BC is the leading cause of cancer death among women, with most patients presenting at advanced stages, where treatment options are limited [3]. The etiology of BC in Sudan has been rarely investigated. A study at Gezira University (1999-2004) found that BC accounted for 456 of 2,233 cancer cases (20.4%) [4]. BC and other malignancies are generally more common in northern Sudan, though significant differences in demographic or hormonal risk factors between regions have not been established [5].

BC diagnosed during pregnancy or within the first year after delivery is traditionally classified as pregnancy-associated breast cancer (PABC). Historically, 0.2%-3.8% of breast malignancies occur during pregnancy [6]. However, recent evidence suggests that BC during pregnancy and postpartum may represent distinct entities, with unique biological characteristics and prognoses [7]. Diagnosing BC postpartum is more common than during pregnancy, though determining the exact onset is challenging [8]. Treating pregnant BC patients requires a delicate balance between optimizing maternal outcomes and minimizing fetal risk [9].

Pathologically, invasive ductal carcinoma (IDC) comprises the majority of breast malignancies diagnosed during pregnancy and lactation [10]. The incidence of inflammatory breast cancer (IBC) is higher in pregnant patients (reported up to 26%) compared to non-pregnant controls [11]. Patients with PABC often present with more advanced disease, including larger tumors, greater lymph node involvement, and a higher likelihood of lymphovascular invasion [8,12]. Hormonally, PABC is frequently associated with estrogen receptor (ER)-negative tumors, with reported rates between 54% and 80% [13,14]. Although ER-negative tumors are more common in younger women in general [15], case-control studies have found them to be even more prevalent in pregnant patients compared to age-matched controls [9,12]. Human epidermal growth factor receptor 2 (HER2) positivity has been reported in 28%-58% of PABC cases [12,16].

This study aimed to describe the demographic, clinical, and pathological characteristics of PABC patients at a major teaching hospital in Sudan.

## Materials and methods

Study design and setting

A retrospective, descriptive, cross-sectional, hospital-based study was conducted at Wad-Madani Teaching Hospital in Gezira State, Sudan, between January and June 2023. The hospital is a major referral center affiliated with the University of Gezira.

Study population and sampling

The study screened records of 2,151 patients diagnosed with BC at the surgical outpatient clinic between January 2017 and December 2021. The target age group was 18-50 years (n = 1,349). After excluding 124 patients who were not sexually active or were postmenopausal, 1,225 eligible patients remained. Among these, 33 patients (2.69%) were identified as having developed symptoms during pregnancy or lactation and formed the final study sample. Due to the limited number of cases (approximately three to five per year), a census of all eligible patients during the study period was included. 

Inclusion and exclusion criteria

Included were females aged 18-50 years with confirmed unilateral or bilateral BC via triple assessment, who developed symptoms during pregnancy or lactation. Patients with a past history of BC, those diagnosed prior to pregnancy, menopausal women, and those not sexually active were excluded.

Variables and data collection

Data were extracted from hospital records using a structured data collection sheet. Variables included patient age, body mass index (BMI), age at menarche, age at first pregnancy, pregnancy/lactation status, clinical presentation, time of symptom discovery, receptor status (ER, PR, and HER2), histopathology report, and risk factors (family history and obesity).

Data analysis

Data were entered and analyzed using IBM SPSS Statistics for Windows, Version 26 (Released 2018; IBM Corp., Armonk, NY, USA). Descriptive statistics were applied. Findings were organized and presented using tables, charts, and figures to ensure clarity and effectiveness.

Ethical considerations

Ethical clearance was obtained from the Sudan Medical Specialization Board, the Gezira State Ministry of Health, and the Surgical Department of Wad-Madani Teaching Hospital. Patient confidentiality was maintained through the use of anonymized data coding.

## Results

Sample characteristics

Of 2,151 BC patients screened, 33 (2.69% of the eligible cohort aged 18-50 years) with PABC were included. The distribution by year of diagnosis was: 2018 (16/33, 48.5%), 2019 and 2020 (7/33, 21.2% each), 2017 (2/33, 6.1%), and 2021 (1/33, 3.0%). Regarding timing, 19 patients (57.6%) were diagnosed during lactation, and 14 (42.4%) during pregnancy.

Clinical presentation

The clinical presentations are detailed in Table 1. The most common presentation was a palpable breast lump (31/33, 93.9%). Other presentations included breast pain (12/33, 36.4%), skin changes or ulceration (10/33, 30.3%), axillary lump (7/33, 21.2%), increased breast size (3/33, 9.1%), and abnormal nipple discharge (2/33, 6.1%). Inflammatory features were present in 11 patients (33.3%).

**Table 1:** Clinical presentation of patients diagnosed with pregnancy-associated breast cancer (PABC) from January 2017 to December 2022 at Wad-Madani Teaching Hospital (n = 33).

Clinical Presentation	Number of Patients, N (%)
Palpable Breast Lump	31 (93.9%)
Breast Pain	12 (36.4%)
Skin Changes/Ulceration	10 (30.3%)
Axillary Lump	7 (21.2%)
Increased Breast Size	3 (9.1%)
Abnormal Nipple Discharge	2 (6.1%)
Inflammatory Features Present	11 (33.3%)

Demographic and clinical characteristics

The demographic and clinical characteristics of the patients are summarized in Table 2. The mean age was 34.2 years (SD ± 5.1). Based on BMI, nine patients (27.3%) were obese, seven (21.2%) were overweight, and 17 (51.5%) had a normal BMI. Ten patients (30.3%) experienced menarche before age 12, and six patients (18.2%) had their first pregnancy after age 35. A positive family history of BC was reported by four patients (12.1%). No other risk factors (hormonal medication, chest radiation, smoking) were identified.

**Table 2:** Demographic and clinical characteristics of patients diagnosed with pregnancy-associated breast cancer (PABC) (n = 33). Note: Some percentages may not sum to 100% due to rounding. BMI: Body Mass Index; BC: Breast Cancer; SD: Standard Deviation

Characteristic	Category/Value	Number of Patients, N (%)
Age (Years)	Mean ± SD	34.2 ± 5.1
BMI Category	Normal (18.5-24.9 kg/m²)	17 (51.5%)
	Overweight (25-29.9 kg/m²)	7 (21.2%)
	Obese (≥30 kg/m²)	9 (27.3%)
Age at Menarche	<12 years	10 (30.3%)
	≥12 years	23 (69.7%)
Age at First Pregnancy	<35 years	27 (81.8%)
	≥35 years	6 (18.2%)
Family History of BC	Positive	4 (12.1%)
	Negative/Unknown	29 (87.9%)
Timing of Diagnosis	During Pregnancy	14 (42.4%)
	During Lactation	19 (57.6%)

Hormonal receptor status

The hormonal receptor status is presented in Table 3. A total of 21 patients (63.6%) were estrogen/progesterone receptor (ER/PR)-negative, while 12 (36.4%) were ER/PR-positive. Regarding human epidermal growth factor-2 (HER2) status, nine patients (27.3%) were positive, and 24 (72.7%) were negative. Four patients (12.1%) were triple-positive (ER/PR/HER2), and 13 (39.4%) were triple-negative. The combination of ER/PR-positive and HER2-negative was observed in 10 patients (30.3%).

**Table 3:** Hormonal receptor status of patients diagnosed with pregnancy-associated breast cancer (PABC) (n = 33). ER: Estrogen Receptor; PR: Progesterone Receptor; HER2: Human Epidermal Growth Factor Receptor 2

Receptor Status	Number of Patients, N (%)
ER/PR Status	
Positive	12 (36.4%)
Negative	21 (63.6%)
HER2 Status	
Positive	9 (27.3%)
Negative	24 (72.7%)
Combined Receptor Status	
Triple Positive (ER+/PR+/HER2+)	4 (12.1%)
Triple Negative (ER-/PR-/HER2-)	13 (39.4%)
ER/PR Positive & HER2 Negative	10 (30.3%)
ER/PR Negative & HER2 Positive	6 (18.2%)

Histopathology

Histopathology reports indicated that IDC was the most common type, found in 28 patients (84.8%). Other types included medullary carcinoma (2/33, 6.1%) and one patient (3.0%) each with papillary carcinoma, squamous cell carcinoma, and malignant phyllodes tumor.

## Discussion

The incidence of PABC in our study was 2.69%, which aligns with the reported global range of 0.2%-3.8% [6,17]. Consistent with other studies [8,17], we found a higher proportion of diagnoses during lactation (57.6%) than during pregnancy (42.4%). The most common clinical presentation was a palpable breast lump (93.9%), consistent with previous reports [17-19]. IBC was present in 33.3% of our patients, a proportion notably higher than the general population estimate of 1.5%-4% [11], underscoring the aggressive presentations often seen in PABC.

Key risk factors identified were obesity (27.3% obese and 21.2% overweight), early menarche (<12 years, 30.3%), and late first pregnancy (≥35 years, 18.2%). These findings are consistent with established BC risk profiles [20-22]. A positive family history was less common (12.1%), consistent with population-based data showing that most BC cases are sporadic [23].

Histopathologically, IDC was predominant (84.8%), mirroring global patterns where IDC constitutes the vast majority of BCs [10,24]. Hormonal receptor analysis revealed a high prevalence of ER/PR-negative tumors (63.6%), a hallmark of PABC reported in other series (54%-80%) [13,14]. HER2 positivity was 27.3%, within the broad reported range (28%-58%) [25]. Notably, 39.4% of our patients had triple-negative BC, a subtype associated with poorer prognosis, highlighting the aggressive biology often encountered in PABC.

Recommendations

The following actions are recommended to improve the management of PABC. First, healthcare providers, particularly those in obstetric and surgical clinics, should receive targeted training to enhance recognition of PABC and to distinguish malignant changes from normal physiological alterations in the breast during pregnancy and lactation. Second, management of patients diagnosed with PABC should involve a multidisciplinary team, including oncologists, surgeons, obstetricians, and radiologists, to optimize maternal treatment while ensuring fetal safety. Third, clinical breast examination should be incorporated as a routine component of antenatal and postnatal care visits due to its safety. Fourth, for patients identified as high-risk, such as those with a strong family history, follow-up should include consideration of radiological imaging, as clinical examination alone has low sensitivity during this period. Finally, for women with a significant family history of BC, discussion and consideration of MRI screening is advised.

Limitations

Several limitations are inherent to the retrospective design of this study. The primary limitation was reliance on hospital records, which resulted in incomplete data for some patients and their exclusion from certain analyses. Additionally, the study did not collect or analyze data on treatment modalities or subsequent clinical outcomes, such as survival or recurrence rates, due to unavailable follow-up information. The single-center design and relatively small sample size, although reflective of the local presentation, may limit the generalizability of the findings to other populations or settings.

## Conclusions

In conclusion, although pregnancy does not inherently worsen the prognosis of BC, diagnostic delay remains a significant challenge in PABC. Physiological changes in the breast during pregnancy and lactation can mask malignant signs, resulting in later-stage presentations, as observed in this cohort. The findings reinforce that a palpable lump is the predominant symptom and that PABC is frequently associated with aggressive features, including inflammatory presentation and triple-negative receptor status. These insights highlight the critical need for heightened clinical awareness, prompt investigation of breast symptoms in pregnant and lactating women, and a structured, multidisciplinary approach to care to improve early detection and outcomes for this patient population.
